# Identification of phloem-associated translatome alterations during leaf development in *Prunus domestica* L.

**DOI:** 10.1038/s41438-018-0092-4

**Published:** 2019-02-01

**Authors:** Tamara D. Collum, Elizabeth Lutton, C. Douglas Raines, Christopher Dardick, James N. Culver

**Affiliations:** 1 Institute for Bioscience and Biotechnology Research, College Park, MD USA; 2 USDA-ARS, Appalachian Fruit Research Laboratory, Kearneysville, WV USA; 3 Department of Plant Science and Landscape Architecture, University of Maryland, College Park, MD USA

**Keywords:** Plant molecular biology, Leaf development

## Abstract

Phloem plays a fundamental role in plants by transporting hormones, nutrients, proteins, RNAs, and carbohydrates essential for plant growth and development. However, the identity of the underlying phloem genes and pathways remain enigmatic especially in agriculturally important perennial crops, in part, due to the technical difficulty of phloem sampling. Here, we used two phloem-specific promoters and a translating ribosome affinity purification (TRAP) strategy to characterize the phloem translatome during leaf development at 2, 4, and 6 weeks post vernalization in plum (*Prunus domestica* L.). Results provide insight into the changing phloem processes that occur during leaf development. These processes included the early activation of DNA replication genes that are likely involved in phloem cell division during leaf expansion, as well as the upregulation of phloem genes associated with sink to source conversion, induction of defense processes, and signaling for reproduction. Combined these results reveal the dynamics of phloem gene expression during leaf development and establish the TRAP system as a powerful tool for studying phloem-specific functions and responses in trees.

## Genetics: the basis of plum plant molecule transport during development

Genes likely involved in DNA replication, immune defenses, stress response, and more occur during development within the plum plant tissue that transports molecules such as nutrients, genetic material, and hormones. The genes underlying this tissue, called phloem, are sparsely understood, inspiring a US team of researchers—led by the University of Maryland’s James Culver—to categorize their activity. Using a technique that “traps” cell-specific genetic material in the process of translation, the team analyzed plum phloem gene expression at 2, 4, and 6 weeks after the induction of leaf development. Finding reduced activity associated with DNA replication activity and an increase in activity associated with immune response, response to nutrients, and transport of sugars among others. These findings could power investigations into phloem processes and how they could be manipulated for enhanced crop production.

## Introduction

In plants, the phloem is the major conduit for the long-distance transport of photoassimilates, phytohormones, small molecules, and macromolecules including RNAs and proteins. This long-distance transport system is vital for plant development and physiology and allows the plant to respond to a diverse array of abiotic and biotic stresses^1–3^. Plant pathogens, such as viruses and some bacteria, can also utilize the phloem to spread systemically throughout a host plant or to be picked up by phloem-feeding insects^4–6^. This makes the phloem a key tissue of interest for investigating host–pathogen interactions, as well as plant development. In recent years, the unique population of mRNAs found in the phloem have been at least partially identified in several plant species including *Arabidopsis thaliana*, melon, potato, grape, rice, and barley^7–15^. Identification of phloem-associated mRNAs has given greater insight into a range of plant physiological processes, such as sucrose loading and systemic acquired resistance (SAR) that occur within phloem tissues^16–18^. However, much information regarding the identity of phloem-associated genes and gene pathways remains to be investigated, especially within perennial plant species.

The phloem is comprised of companion cells (CCs) and sieve elements (SEs) surrounded by support cells including bundle sheath and phloem parenchyma^3,19^. At maturity, the anucleate SEs are dependent on adjacent CCs for genetic and metabolic capabilities. Together these cells form a pressurized system in which the phloem translocation stream flows from source to sink tissues. Isolating phloem contents from this pressurized system can be technically challenging. The majority of plants where phloem mRNAs have been identified are those that naturally exude sap upon wounding. For plant species that do not naturally exude sap, the addition of the chelator EDTA has been used to facilitate phloem bleeding^20–22^. However, this method has a risk of introducing contaminants to the phloem sap due to the damage of neighboring cells^17,23–25^. Other methods such as phloem collection from insect stylectomy or phloem microdissection can be technically difficult and costly to establish. In this study, we used a translating ribosome affinity purification (TRAP) approach to isolate mRNAs from phloem tissues^26^. In this method, a tagged ribosomal protein is expressed from phloem-specific promoters. This allows for the immunopurification of ribosome–mRNA complexes specifically from phloem tissues without disruption of the pressurized phloem system prior to mRNA harvesting. mRNAs eluted from tagged ribosomes (termed the translatome) are then identified by RNA-seq. A further advantage of this approach is that in contrast to total cellular mRNAs (termed the transcriptome) mRNAs associated with ribosomes are likely in the process of translation and thus more directly impact cell physiology.

In temperate crops such as fruit trees, phloem tissues are renewed annually in stems and newly developing leaves following a period of winter dormancy. Together with the xylem, phloem tissues establish sink to source movement of photoassimilates that drive carbohydrate partitioning. These fundamental functions are critical for numerous agronomic characteristics such as growth rate, fruit size, and fruit sweetness. For perennial tree crops, there exists little information regarding the identity of phloem-expressed gene products within the vascular tissues. Previous studies using phloem exudates derived by excision or aphid stylectomies have identified a number of proteins and RNAs present in the SEs of these woody perennials^22,27^. However, mature SEs are generally considered to be incapable of transcription and translation^28–31^. Thus, previously identified SE components are likely to be part of the translocation stream and in transit within the vasculature.

To better investigate phloem processes, specifically those that occur during leaf development and expansion, we have used a tissue-specific translatome approach to identify ribosome-associated mRNAs that are likely being actively translated within the leaf phloem of *Prunus domestica* L. Results from this study provide understanding into the changing cellular processes that occur during leaf phloem development, as well as the identity of specific genes associated with these processes. The utility of the TRAP system for studying phloem functions in perennial crops is also discussed.

## Results

### Isolation of translating ribosomes from plum

**Fig. 1:**
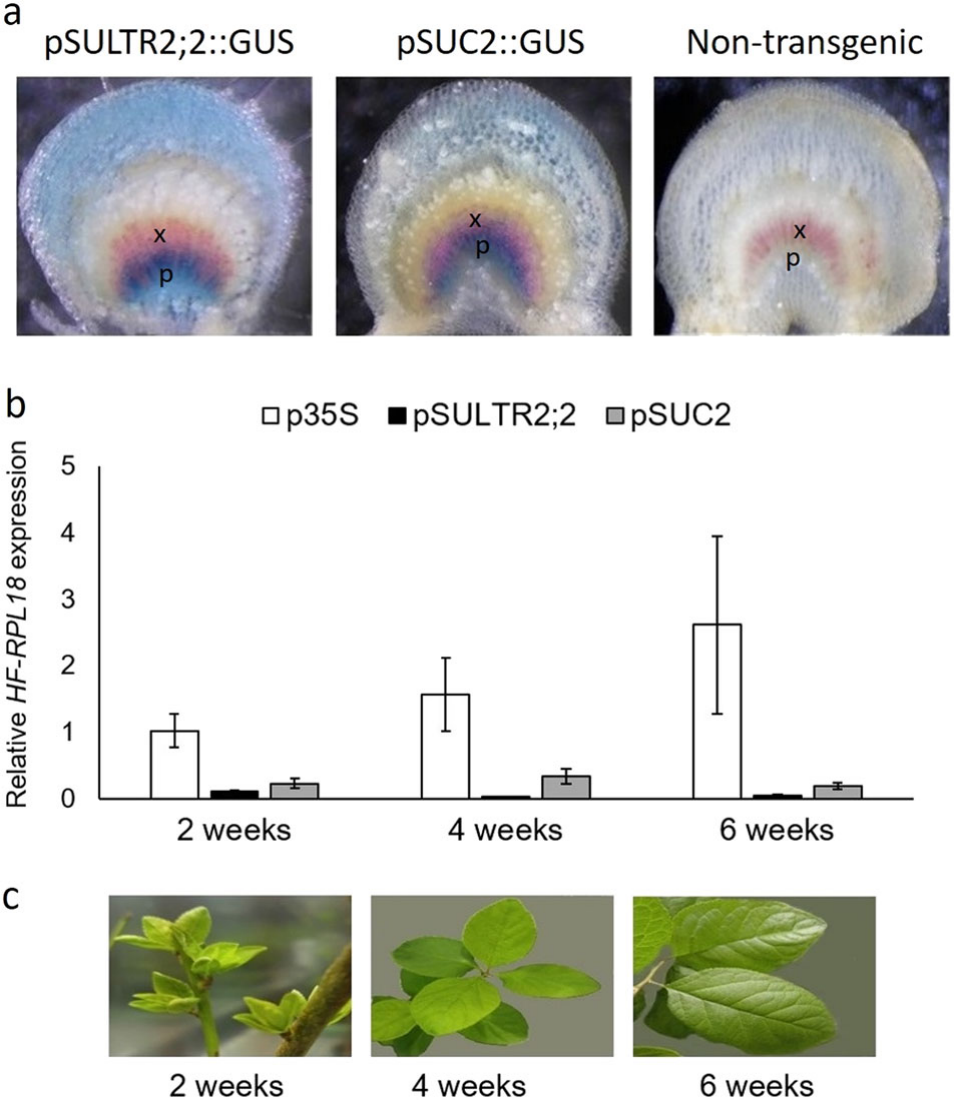
*Prunus domestica* L. promoter:HF-RPL18 transgenic plants. **a** Histochemical analysis of Arabidopsis pSUC2 and pSULTR2;2 promoters in transgenic plums visualized by GUS staining in mid-vein cross sections. Phloroglucinol was used to stain xylem red. x xylem, p phloem. **b** Relative HF-RPL18 transgene expression in leaves. Quantitative RT-PCR analysis was performed with a primer set specific to HF-RPL18 and 18S rRNA was used as the internal control. Bars represent the mean of three biological replicates ± standard error. **c** Representative photographs of leaves collected at 2, 4, and 6 weeks post vernalization

To identify phloem-specific mRNAs associated with ribosomes in plum, we generated transgenic *Prunus domestica* L. that express the *Arabidopsis thaliana* ribosomal protein L18 (RPL18) tagged with a His_6_-FLAG (HF) dual-epitope driven by either one of two phloem-specific promoters, pSUC2 or pSULTR2;2 that were acquired from *A. thaliana*, as well as the more ubiquitously expressed CaMV 35S promoter. *A. thaliana* RPL18 shares 87% amino-acid identity and 95% similarity with plum RPL18 (Fig. S1). *A. thaliana* pSUC2 has been previously shown to be expressed specifically in phloem vascular tissues in many plant species including pear, lime, and sweet orange trees^32–34^, whereas *A. thaliana* pSULTR2;2 has been shown to be expressed in phloem vascular tissues in *A. thaliana* and *Nicotiana benthamiana*^15,35^. pSUC2 and pSULTR2;2 have also been shown to have slightly different expression patterns with pSUC2 driving expression predominately in shoot CCs, whereas pSULTR2;2 expresses in both shoot CCs and bundle sheath cells^15,35–38^. To confirm the phloem-specific expression of pSUC2 and pSUTLR2;2 in *Prunus domestica* L., we created pSUC2::GUS and pSULTR2;2::GUS reporter lines. We found that GUS expression was observed in phloem tissues in plum leaves when driven by either pSUC2 or pSUTLR2;2 promoters but not in non-transgenic control plants (Fig. 1a). Consistent with previous reported results, we saw broader expression of GUS when driven by the pSULTR2;2 promoter compared with pSUC2.

**Fig. 2:**
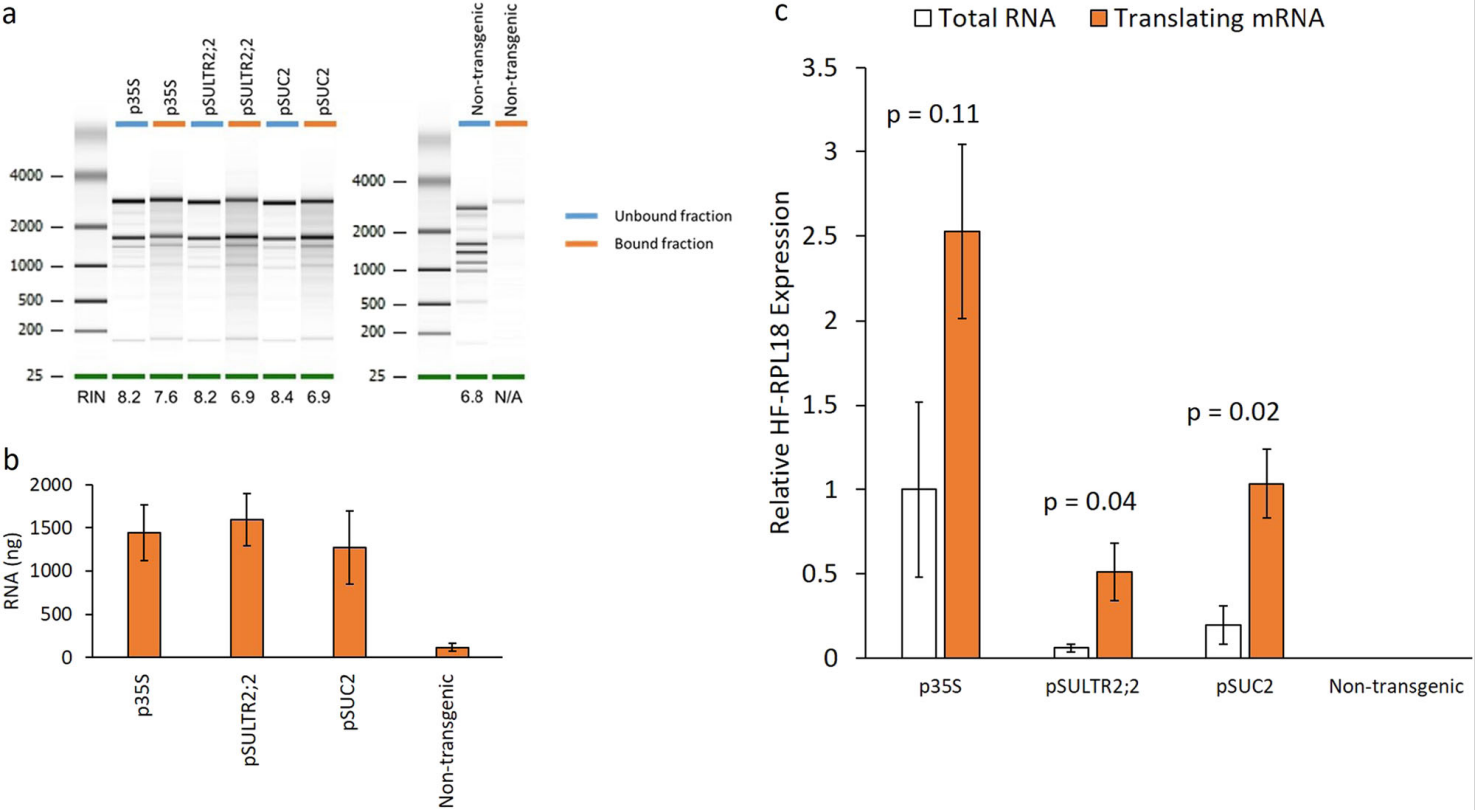
Isolation of translatome RNA. **a** Representative Agilent 2100 bioanalyzer gel-like image showing RNA isolated after incubation with Anti-FLAG magnetic beads taken from the unbound (blue labels) or bound (orange labels) fraction. High-quality RNA was successfully recovered in the bound fraction for all translatome HF-RPL18 plant lines, but not from non-transgenic controls. **b** Quantification of RNA recovered from the bound fraction. RNA was measured on a NanoDrop 2000 and is shown in nanograms. Bars represent the mean of four biological replicates ± standard error. **c** Relative HF-RPL18 transcript accumulation detected in total RNA (white bars) and translating mRNA samples (orange bars). Bars represent the mean of three biological replicates ± standard error. All samples are normalized to p35S total RNA

To confirm expression of HF-RPL18, leaf tissue was collected from plum trees at 2, 4, and 6 weeks post vernalization. A vernalization treatment of 60 days was used to mimic the period of winter dormancy. This chilling period is required to initiate normal bud break and new leaf growth after trees are exposed to a period of favorable temperatures. Phloem tissues are renewed annually after dormancy in newly developing leaves. We chose to sample leaves every 2 weeks after dormancy to identify phloem-specific genes and pathways that contribute to this process. Quantitative RT-PCR (qRT-PCR) was used to monitor expression of the HF-RPL18 transcript at each time point. We observed the highest expression level from the p35S promoter, followed by pSUC2 then pSULTR2;2 (Fig. 1b). There was no significant difference in expression of HF-RPL18 between time points for p35S and pSUC2, but pSULTR2;2 had fourfold and twofold higher expression at 2 weeks than 4 weeks and 6 weeks (*p*-values = 0.003 and 0.024, respectively). To purify polysome–mRNA complexes, leaf extracts prepared with a polysome extraction buffer (PEB) were loaded onto a sucrose cushion and ultracentrifugation was used to pellet the polysome fraction. Any HF-RPL18 proteins that are not incorporated into ribosome complexes will remain in the supernatant and be removed. Anti-FLAG magnetic beads were added to the resuspended polysome fraction to capture tagged polysome–mRNA complexes. We could recover high-quality RNA from all plant lines expressing HF-RPL18 but not from non-transgenic controls (Fig. 2). qRT-PCR was used to determine HF-RPL18 expression in mRNAs isolated from polysomes compared with total RNAs. We found HF-RPL18 expression significantly increased 8.5 to 5.3-fold in pSULTR2;2 and pSUC2 translating mRNA compared with total RNA (*p* < 0.05) (Fig. 2c). In contrast, HF-RPL18 expression in p35S translating mRNA samples had a smaller 2.5-fold increase that was not statistically significant (*p* = 0.11). These data are consistent with phloem-specific expression of HF-RPL18 in pSULTR2;2 and pSUC2 transgenic plant lines and successful enrichment of tissue-specific transcripts. Together these results indicate that *A. thaliana*-derived HF-RPL18 is expressed in the predicted tissue-specific fashion in plum and can be used to capture mRNAs from translating ribosomes.

### Phloem-associated translatome analysis

To characterize phloem-specific translatome profiles during post-dormancy leaf development, RNA was isolated from tagged polysome complexes of leaf extracts at 2, 4, and 6 weeks post vernalization (Fig. 1c). Three biological replicates, each comprised of leaf tissue collected from individual clonal trees of the same lines, were isolated for each promoter at each time point to generate a total of 27 libraries. Forty-three to 63 million paired-end reads were generated for each sample (Table S1). Paired-end reads were mapped with CLC Genomics Workbench v. 10.0.1 using the closely related *Prunus persica* genome version 2.0 as a reference^39^. Principal component analysis showed that samples were separated primarily on principal component 1 (29.7%) according to age and to a more limited extent on principal component 2 (17.5%) by construct (Fig. S2). As a control, we additionally sequenced ribosome-associated RNAs from non-transgenic leaves collected at 2 weeks. Ultracentrifugation was used to pellet the ribosome fraction and RNA was isolated from the resuspended pellet, but these non-transgenic samples were not incubated with anti-FLAG magnetic beads as they do not express a tagged protein. Non-transgenic samples separated on principal component 1 (37.4%) from the other 2-week samples (Fig. S3a). Differential gene expression analysis was done using CLC Genomics Workbench RNA-seq tool using a threshold of fold change > 2 and false discovery rate (FDR) *p*-value < 0.05. Non-transgenic samples had 6137, 6781, and 7297 differentially expressed genes (DEGs) when compared with p35S, pSUC2, and pSULTR2;2 samples respectively (Fig. S3b). We expected non-transgenic samples isolated by ultracentrifugation to be similar to p35S samples, as they both capture ribosomes for nearly all cell types. Although as expected, p35S did have the least number of DEGs when compared with non-transgenic controls, p35S samples were more similar to pSUC2 (700 DEGs) and pSULTR2;2 (686 DEGs) samples than non-transgenic controls (6137 DEGs). We concluded that p35S samples that had been through the immunopurification step were a more suitable control than non-transgenic ribosome-associated RNAs collected after ultracentrifugation.

After filtering and trimming, 72–90% of reads mapped to the reference genome, except for 4-week sample pSUC2-3, which had 43% of reads mapping to the genome. Of the reads that mapped, 66–94% mapped to exons, except for all three 4-week p35S samples and one 2-week p35S sample in which 31–53% of reads mapped to exons. Reads that did not map to exons mapped primarily to intergenic regions (Table S1). To our knowledge, *Prunus domestica* L. mapping rates to *Prunus persica* have not been previously published. However, percentages of mapped reads in this study are consistent with previous reports for other Prunus species. Peach-derived transcripts mapped to the *Prunus persica* reference genome have been reported at rates of 83–94%^40,41^. Although apricot-derived transcripts mapped to the *Prunus persica* reference genome at a lower rate of 64–72%^42^. Reads that did not map to exons could represent novel splice junctions or unannotated transcripts. In all samples, reads mapping to the Arabidopsis RPL18 His-FLAG tag sequence were present. AtRPL18 transcript levels were relatively consistent across biological replicates and there were no significant differences in reads mapping to the RPL18 His-FLAG tag sequence between time points (Table S1, Fig. S4).

To identify genes significantly enriched in phloem tissues, we compared pSUC2 or pSULTR2;2 samples to p35S samples for each time point. p35S is expressed in almost all tissue types including phloem, however, we would expect that phloem-derived mRNAs would be only a small fraction of all translating mRNAs isolated from p35S trees. Therefore, we would expect transcripts expressed predominately in phloem tissues to have significantly higher expression in pSUC2 and pSULTR2;2 samples when compared with p35S. Filtering transcripts identified in pSUC2 and pSULTR2;2 samples against p35S also compensates for any background non-phloem expression derived from the phloem promoters. Differential gene expression analysis was done using CLC Genomics Workbench RNA-seq tool using a threshold of fold change > 2 and FDR *p*-value < 0.05. A total of 1798 genes were identified as phloem enriched in the pSUC2 translatome for at least one time point, whereas 2149 genes were identified in the pSULTR2;2 translatome (Table S2).

Phloem-associated translatomes have only been previously characterized using TRAP in *A. thaliana* and *N. benthamiana*^15,35^. The number of plum phloem-associated genes identified in this study are consistent with results from *N. benthamiana* where 2308 genes were enriched in the pSUC2 translatome and 1236 genes were enriched in the pSULTR2;2 translatome^15^. Although in Arabidopsis, phloem translatomes included only 204–798 enriched genes^15,35^. To compare the plum and *N. benthamaina* phloem-enriched gene lists, we used the Arabidopsis best BLAST match for each gene. For plum, a total of 1740 unique genes were identified as associated with phloem pSUC2 or pSULTR2;2 translatomes, as many plum genes best BLAST match was the same Arabidopsis gene. A total of 368/1740 (21%) of these genes were previously identified as phloem enriched in *N. benthamiana*^15^. In contrast, 36/1740 (2%) of identified plum genes were also associated with phloem translatomes in Arabidopsis. However, there was greater overlap between plum phloem translatomes and the phloem transcriptome isolated from Arabidopsis phloem exudates with 174/1740 (10%) overlap^9^. Even when comparing the same species, phloem studies done by different groups have had only 8–22% overlap^9,35,43^. The low percent of overlap can be explained by differences in phloem isolation techniques and sampling times, which ranged from 1 week to 10 weeks. Thus, up to 21% overlap with previous identified phloem-associated transcripts is consistent with isolation of phloem tissues from plum.

To further validate that we had successfully isolated phloem-specific mRNAs, we first searched for known phloem-expressed genes. Eighty-eight Arabidopsis genes are annotated as associated with phloem tissues (PO:0005417). We found 16/88 (18%) of phloem-associated genes enriched in pSUC2 and/or pSULTR2;2 translatomes in our plum dataset (Table S3). In contrast, only 4/78 (5%) genes annotated as associated with xylem (PO:0005352) were enriched in our plum phloem dataset (Table S4). This is consistent with previous Arabidopsis phloem studies where 25% of phloem annotated genes were found in in phloem exudate or laser microdissection pressure catapulting derived phloem tissue compared with 13% of xylem annotated genes^9^. Taken together, the identification of known phloem-associated genes as significantly enriched in our phloem translatome dataset suggests the successful isolation of phloem-associated mRNAs from the plum translatome.

### Identification of phloem-associated translatome alterations during leaf development

**Fig. 3:**
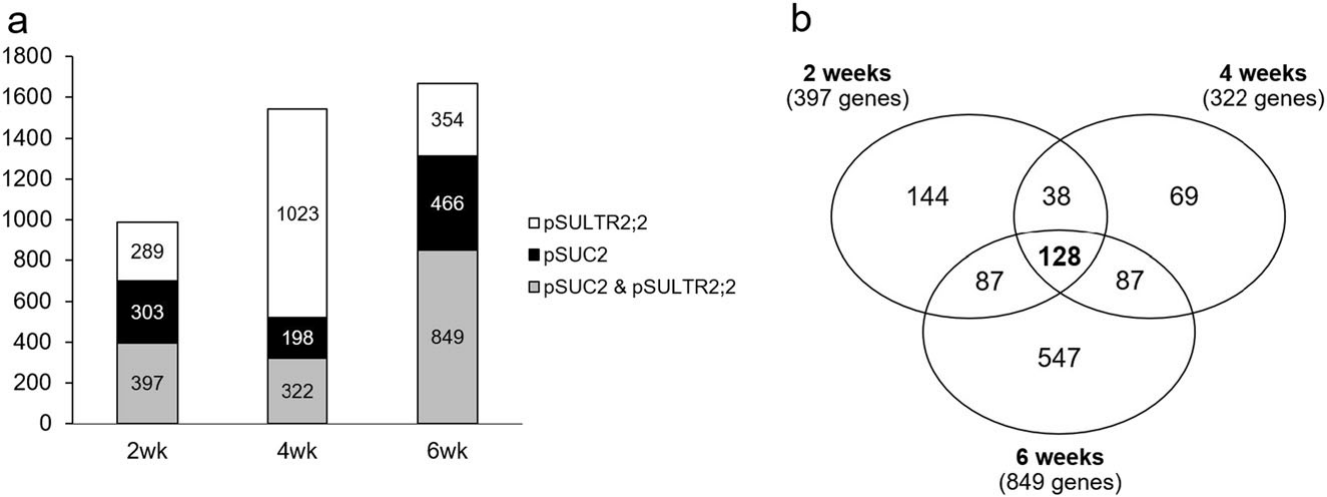
Phloem-enriched genes. **a** Total number of genes with > 2-fold expression that are unique or shared by pSUC2 or pSULTR2;2 phloem translatomes compared with p35S translatome at 2, 4, and 6 weeks post vernalization. **b** Number of unique and shared phloem-enriched genes across time points. Phloem-enriched genes were defined as > 2-fold expression in both pSUC2 and pSULTR2;2 phloem translatomes compared with p35S translatome

The highest number of phloem-enriched genes were found at the 6-week time point with a total of 1669 genes enriched in either the pSUC2 or pSULTR2;2 translatomes. Of these genes, 849 (51%) were significantly enriched in both translatomes. At 4 weeks, 1543 genes were identified as phloem enriched, with 322 (21%) significantly enriched in both phloem translatomes. While at 2 weeks, we found a total of 989 phloem-enriched genes with 397 (40%) significantly enriched in both phloem translatomes (Fig. 3a, Table S5). Twenty-one to 51% overlap between pSUC2 and pSULTR2;2 phloem translatomes is consistent with previous studies and is likely due to pSUC2 driving expression predominately in shoot CCs, whereas pSULTR2;2 expresses in both shoot CCs and bundle sheath cells^15,35–38^. Next, we chose to focus on the genes that were found to be significantly enriched in both pSUC2 and pSULTR2;2 translatomes for further analysis (Table S5). Of the 1100 genes enriched in both phloem translatomes, 128 (11.64%) genes were significantly enriched at all time points tested (Fig. 3b, Table S6). Among others, 144 were enriched only at the early 2-week time point, 69 were uniquely enriched at 4 weeks, and 547 genes were enriched only at 6 weeks (Fig. 3b). Gene ontology (GO) enrichment analysis using the best *A. thaliana* BLAST match for each gene, found that the plum phloem was enriched for genes associated with response to stress, defense responses, and protein phosphorylation at all time points tested (Fig. S5). At the 2-week time point, additional GO terms associated with defense and immune system responses were significantly enriched, while at 6-week genes associated with reproduction and transmembrane transport were uniquely enriched (Fig. S5).

**Fig. 4:**
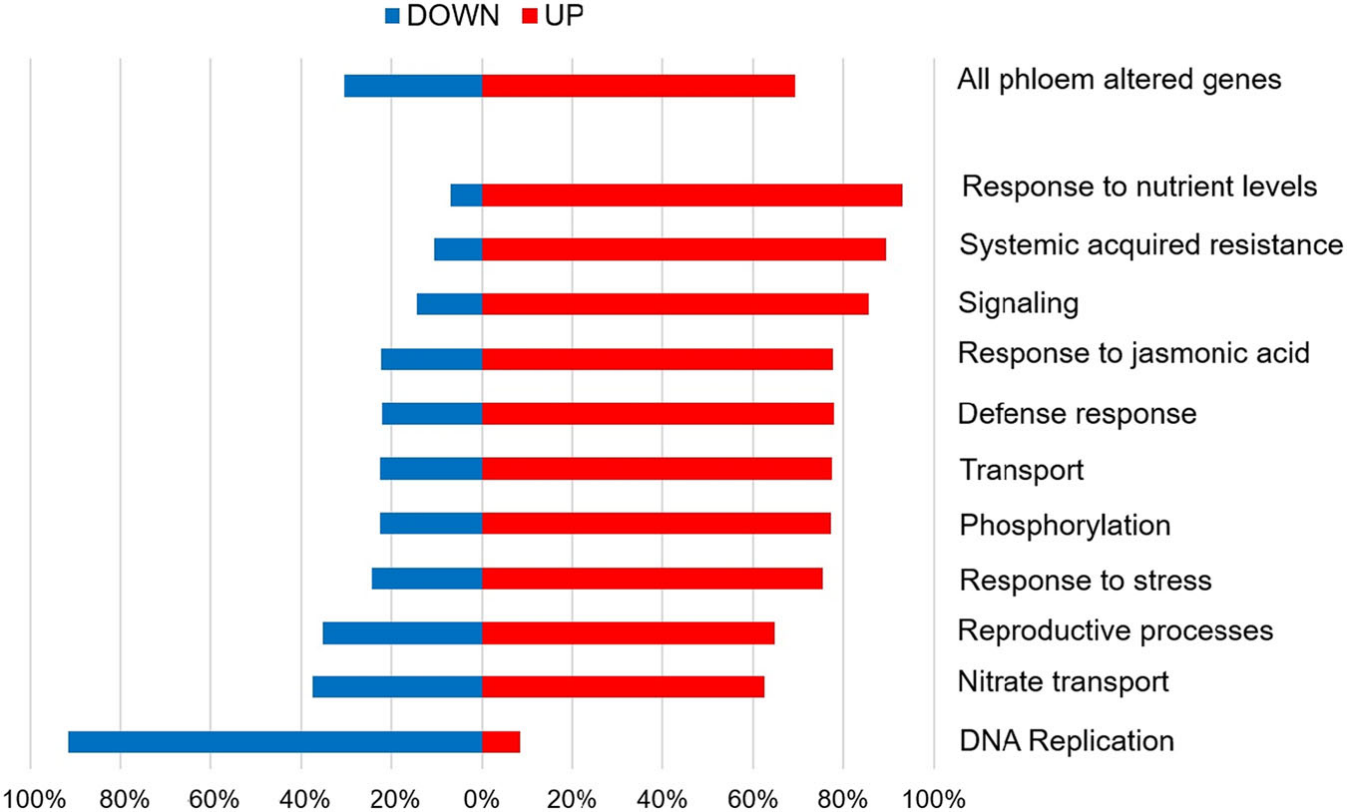
Phloem-enriched gene categories altered over development. A subset of gene ontology (GO) biological process terms overrepresented in phloem-enriched genes that are altered over the 2- to 6-week sampled time frame. Bars represent the percentage of genes in each category that are downregulated (blue) or upregulated (red). All significantly overrepresented GO biological process terms are listed in Table S7

**Fig. 5:**
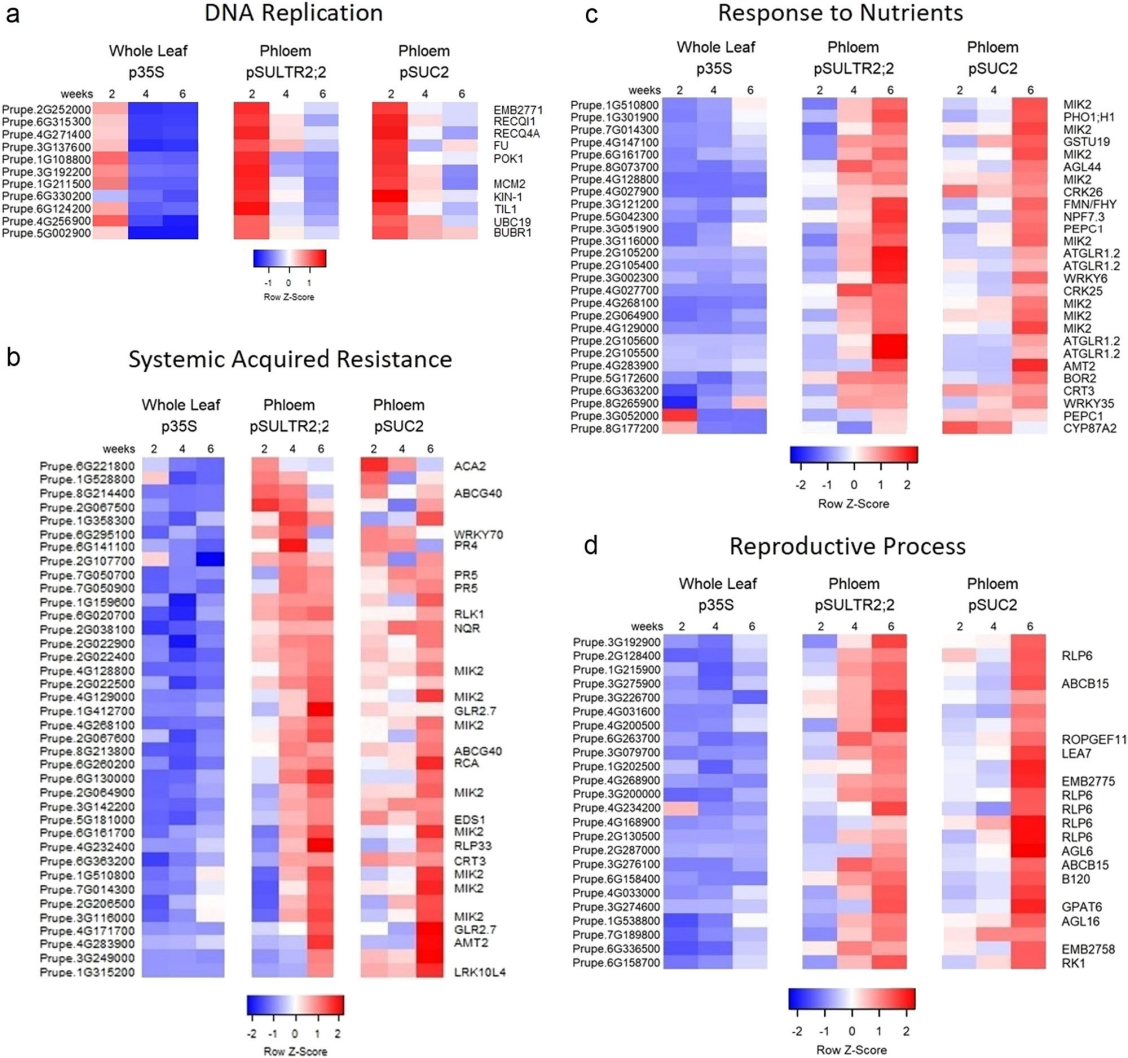
Heatmaps of phloem-enriched gene expression over development. Heatmaps showing changes to genes involved in **a** DNA replication, **b** systemic acquired resistance, **c** response to nutrients, and **d** reproduction in *Prunus domestica* L. leaves 2, 4, and 6 weeks post vernalization. Mean FPKM values from three biological replicates are shown on a log2 scale with z-scaling by row. Gene names from *A. thaliana* best BLAST matches are shown to the right of all heatmaps

To further analyze how the phloem translatome is altered during development, we looked for those genes that were differentially expressed between leaf developmental time points. Differential gene expression analysis was done using CLC Genomics Workbench RNA-seq tool using a threshold of absolute value fold change > 2 and FDR *p*-value < 0.05. Of the 1100 genes enriched in both pSUC2 and pSULTR2;2 translatomes, 161/1100 (15%) were significantly downregulated over time in one or both phloem translatomes, 366/1100 (33%) were significantly upregulated, and the remaining 573/1100 (52%) did not significantly change expression over time (Table S5). We identified GO terms that were significantly overrepresented among the 527 phloem genes altered during leaf development and plotted the percentage of genes that were upregulated or downregulated for each GO term (Fig. 4, Table S7). DNA replication was the only GO term where the majority of genes were downregulated during leaf development. Of the 11 downregulated DNA replication genes identified, all displayed a reduction between the 2- and 4-week sampling times (Fig. 5a).

Surprisingly, the defense response category was the most significantly overrepresented GO term (FDR *p*-value 1.40e–13) among the 527 phloem genes altered during leaf development (Table S7). Seventy-eight percent (92/118) of defense-related genes were upregulated over the course of leaf development (Fig. 4). Identified defense genes included 38 genes that are involved in SAR with 79% (30/38) showing the highest expression at 6 weeks in phloem translatomes (Fig. 5b). Additional GO categories where the majority of genes were upregulated over time included response to nutrient levels and reproductive processes (Figs. 4, 5). Response to nutrient levels had the highest percentage of upregulated genes over time at 93% (27/29 genes) (Fig. 4).

### Validation of selected transcripts

To further confirm the results obtained by RNA-seq, 12 genes were selected for analysis using qRT-PCR. Eight of these genes were identified as phloem enriched in both phloem translatomes at all time points tested, two had higher expression in non-phloem tissues, and two had similar expression levels in all samples. Three additional translatome biological replicates were collected from a different set of trees at the 2-week post vernalization time point. qRT-PCR analysis mirrored the RNA-seq translatome study (Fig. S6). Combined these results indicate phloem-enriched mRNAs identified by TRAP-seq in this study are reproducible in additional biological replicates.

## Discussion

Vascular phloem represents one of the most important and specialized tissues in higher plants. Yet our understanding of the phloem’s transcriptional and translational composition is generally limited, particularly for plant systems such as woody perennials. The goal of this study was to assess the phloem translatome responses during leaf development in plum trees. Here we used a new molecular approach to express a tagged ribosomal protein (AtRPL18) from two phloem-specific promoters, pSUC2 and pSULTR2;2, in order to capture mRNAs from phloem tissues. This approach allows for the isolation of phloem-specific mRNAs without having to physically separate phloem from surrounding tissues, which is extremely technically difficult. Another key advantage of this approach is that mRNAs that are associated with ribosomes are likely being translated and thus have a direct impact on cellular function.

In plum trees, phloem is renewed annually in developing leaves after a period of winter dormancy. Isolation of phloem-associated translating mRNAs during new leaf growth following dormancy allowed us to identify specific phloem-associated genes and their corresponding cellular processes that show differential regulation over a range of leaf developmental stages. Combined these findings provide a unique resource for understanding a range of phloem processes that include sink to source transition, plant defense, and the onset of reproduction. Knowledge into these processes also represents a first step toward future efforts to manipulate phloem processes for enhanced crop production and disease resistance.

Specific findings from these studies included a range of translational alterations, with many DEGs associated with specific leaf developmental and response processes. For example, genes involved in DNA replication represented the only category that was consistently downregulated over successive developmental time points. This was also the GO term that was most significantly overrepresented among all downregulated phloem-enriched genes. These genes included predicted plum homologs of the Arabidopsis *MCM2*, *POK1*, and *UBC19*, all of which are genes shown to be expressed primarily in actively dividing cells^44–48^. High expression of DNA replication genes during early leaf development in both phloem and non-phloem tissues is consistent with the transition from early rapid cellular division to later growth stages where cell division slows and growth predominantly occurs via cellular expansion. Interestingly, the downregulation of these genes at 4 and 6 weeks was significantly greater in p35S whole leaf translatome samples than in the pSUC2 and pSULTR2;2, phloem-associated translatomes, suggesting that phloem remains active for cell division even after the leaf reaches maturity.

The majority of phloem-enriched genes that were altered throughout the course of leaf development were upregulated. Among these genes, the GO term most significantly overrepresented was defense response. Among phloem altered defense genes, 38 genes were identified as involved in SAR. We found 34/38 of SAR genes were upregulated over the course of development with the majority (30/34) showing their highest levels of expression in the phloem at 6 weeks. Genes with the highest expression at 6 weeks included predicted homologs of EDS1, RLP33, and RLK1. An additional four genes had peak expression levels at the 4-week time point and included homologs of pathogenesis related 4 (PR4) and PR5. In contrast, a predicted homolog of WRKY70 was one of only four SAR genes that was downregulated over the course of leaf development in both phloem translatomes. WRKY70 showed high expression levels at 2 weeks and 4 weeks with reduced expression at 6 weeks. WRKY70 has been recently shown to directly repress SARD1, a positive regulator of salicylic acid synthesis, in an Arabidopsis protoplast system and has been reported as an important node for salicylic acid and jasmonic acid signaling in plant defense responses^49,50^. Thus, the downregulation of WRKY70 observed in the phloem could contribute to increased salicylic acid synthesis in mature plum leaves. Salicylic acid accumulation in mature leaves has been previously described and associated with age-related resistance to pathogens in Arabidopsis^51–53^. This is also consistent with the observed upregulation of the majority of SAR genes in plum phloem translatomes at 6 weeks, suggesting that the phloem within mature leaves contributes significantly to the activation of defense responses.

Within the phloem the GO category with the greatest percentage of upregulated genes at 93% (27/29) was response to nutrients. This category includes genes involved in the transport of essential elements involved in growth and development, such as homologs of nitrate and phosphate transport genes of NRT1/ PTR FAMILY 7.3, AGL44, and PHO1;H1. The upregulation of these genes within the leaf vasculature within mature leaf tissues is consistent with its transition to source tissue with significant nutrient import and export functions. Similarly, 65% (33/51) of genes categorized as involved in reproductive processes were upregulated during leaf development, even though the plum trees were still in a juvenile state and did not flower. This included homologs of Arabidopsis AGL6 and AGL16, which encode MADS box transcription factors involved in flowering and transition to a reproductive phase in Arabidopsis^54,55^. However, the predicted FT gene homolog in plum is not significantly enriched in phloem tissues at any time point. Thus, juvenile trees appear to be primed for flowering but lack the FT trigger needed to initiate the process. Collectively, these findings suggest there is a significant transition in the phloem translatome that occurs upon leaf maturation that closely represents the metabolic and developmental transitions associated with sink to source transition and reproduction status.

The majority of phloem-enriched genes (53%) did not significantly change expression levels across the leaf developmental stages we tested. Seventy-four of these genes were significantly enriched in both phloem translatomes compared with p35S at all three time points. Included in this list were several kinases including a homolog of CRK10, which has been shown to be phloem mobile in Arabidopsis^14^. There were also genes associated with structural components of the phloem including homologs of Arabidopsis phloem protein 2-A1 and sieve-element occlusion-related 1. As expected, we found genes involved in the biosynthesis and transport of sugars were highly expressed in phloem tissues at all time points. Additionally, a predicted homolog of PHOSPHATE 2 was also identified in this gene set. In Arabidopsis, PHO2 is involved in the regulation of phosphate transport and has been shown to be expressed in the vasculature^56,57^. Thus, genes in this group that are similarly expressed and phloem enriched at all time points may represent genes involved in general phloem housekeeping functions or transport of nutrients needed throughout development.

In this study, TRAP was successfully used to capture phloem-specific mRNAs at three different developmental stages in plum leaves. The creation of transgenic translatome plant lines in plum and the adaptation of the TRAP method for use in fruit trees provides a powerful set of tools that can be used to identify changes to mRNAs in specific tissue types or at certain temporal stages. In *A. thaliana*, TRAP has been used to compare translatomes during developmental stages^58^ and in response to many abiotic and biotic stresses including low oxygen^35,59^, cold stress^60^, and pathogens^15,61,62^. Results from this study have identified specific expressed genes and processes that uniquely represent phloem responses during leaf development. These findings provide novel candidate genes and pathways that can be used in future studies aimed at improving fruit tree production.

## Materials and methods

### Plant material, transformations, and growth conditions

For the creation of transgenic plant lines, p35S::HF-RPL18, pSUC2::HF-RPL18, and pSULTR2;2::HF-RPL18 translatome constructs were kindly provided by Dr. J. Bailey-Serres, University of California, Riverside, CA, USA^35^. GUS expression constructs were created as described previously^15^. Briefly, upstream promoter sequences covering 2-kb upstream of the *A. thaliana* pSUC2 and pSULTR2;2 open reading frames were cloned into pBI101.1 (Clontech, Mountain View, CA, USA) in front of the GUS reporter open reading frame via primer-generated *Sal*1 and *Bam*H1 restriction sites to create pSUC2::GUS and pSULTR2;2:GUS. Promoter::HF-RPL18 and promoter::GUS vectors were transformed into the Agrobacterium tumefaciens strain GV3101 and used for Agrobacterium-mediated transformation of plum (*Prunus domestica* L.) hypocotyl slices as previously described^63^. Hypocotyls were dissected from seeds of the open-pollinated cultivars “President” and “Stanley”. A minimum of seven independent transgenic lines for each construct were obtained and selected for using Kanamycin, propagated via tissue culture, rooted, and transplanted to soil. Transgene insertions were confirmed by quantitative RT-PCR (qRT-PCR) using primers 5ʹ-ATTTACAATTACCATGGGACATCAC-3ʹ and 5ʹ-CACCACCTCCCTTATCATCATC-3ʹ specific for the His_6_-FLAG-RPL18 transcript. All plum trees were maintained in a growth chamber at 25 °C, 75% humidity, with a 12-h photoperiod. After 12 weeks, plum leaves were stripped and plants were kept at 8 °C for a 60-day vernalization period before being moved back to the growth chamber.

### GUS staining

Leaf and stem tissues were hand dissected (1–2 mm thick) with a razor blade then placed into 2 ml conical tubes filled with cold acetone on ice and incubated. The acetone from each sample was removed and the samples equilibrated with staining buffer^64^. Equilibrated samples were then developed in reaction buffer containing 2 mM of 5-bromo-4-chloro-3-indolyl-beta-d-glucuronic acid, overnight at 37 °C. Developed samples were rinsed in progressive concentrations of ethanol (30 min each at 20, 50, and 70%). Final treatments in 95% ethanol heated to 65 °C at 5- to 10-min increments was performed until the chlorophyll was fully removed. The samples were then observed and photographed in ethanol under a Zeiss Axiozoom microscope (Thornwood, NY, USA) fitted with a color camera.

### Translating ribosomes affinity purification

Isolation of polysomes from non-transgenic or promoter::HF-RPL18 lines was done as previously described^26,35,58^. Briefly, frozen leaf tissue was homogenized in PEB (200 mM Tris-HCl, pH 9.0, 200 mM KCl, 25 mM ethylene glycol tetraacetic acid (EGTA) pH 8.0, 35 mM MgCl_2_, 1% (v/v) octylphenyl-polyethylene glycol (Igepal CA-630), 1% (v/v) polyoxyethylene 10 tridecyl ether, 1% (v/v) sodium deoxycholate, 5 mM dithiothereitol (DTT), 1 mM PMSF, 50 μg/mL cycloheximide, 50 μg/mL chloramphenicol, 0.5 mg/mL heparin) using 10 mL PEB per 5 g of tissue. The supernatant was loaded onto an 8 mL 1.6 M sucrose cushion. Samples were centrifuged at 170,000 *g* for 18 h at 4 °C to pellet the polysomes, which were then resuspended in 1 mL of PEB. For non-transgenic controls, RNA was isolated from the resuspended pellet using the Qiagen RNeasy kit (Qiagen, Valencia, CA, USA) according to the manufacturer's instructions.

Immunopurification of polysomes from promoter::HF-RPL18 plants was done as described^26,35^ with minor modifications. Specifically, anti-FLAG magnetic beads, 50 μL (Sigma Chemical Company, St. Louis, MO, USA) were added to the resuspended pellet and incubated at 4 °C overnight with gentle rocking. The beads were recovered using a magnet and washed four times for 5 min with 1 mL of wash buffer (200 mM Tris-HCl, pH 9.0, 200 mM KCl, 25 mM EGTA, 35 mM MgCl_2_, 5 mM DTT, 50 μg/mL cycloheximide, 50 μg/mL chloramphenicol). The complexes were eluted by treatment of the magnetic beads with 100 μL of Elution Buffer (100 μL wash buffer, 10 μL of 5 mg/mL FLAG3 peptide—Sigma Chemical Company, St. Louis, MO USA, 0.5 μL of 2 U/mL RNAse OUT—Thermo Fisher Scientific Cleveland, OH, USA). RLT buffer plus 2-mercaptoethanol from the Qiagen RNeasy kit (Qiagen, Valencia, CA, USA) was added to the eluted complexes. A 0.5 × volume of 100% ethanol was then added and the sample transferred to Qiagen RNeasy columns. Washes and RNA elutions were performed according to the manufacturer’s instructions. Isolated RNA was measured on a NanoDrop 2000 and with the Qubit RNA Assay Kit (Thermo Fisher Scientific, Cleveland, OH, USA). Quality of the RNA was confirmed using a RNA ScreenTape assay (Agilent Technologies, Palo Alto, CA, USA).

### RNA sequencing and analysis

Library preparation and paired-end sequencing on the Illumina HiSeq system was done by Genewiz (South Plainfield, NJ, USA). Reads that passed Illumina’s quality control filters were further processed. Reads were mapped to the Prunus persica v2.0 genome^39^. The International Peach Genome Initiative 2013, https://www.rosaceae.org/species/prunus_persica/genome_v2.0.a1 using the CLC Genomics Workbench v10.0.1 RNA-seq analysis tool and default parameters (Mismatch cost 2, Insertion cost 3, Deletion Cost 3, Length fraction 0.8, Similarity fraction 0.8, Max hits for a read 10—CLC Bio, Aarhus, Denmark) (Table S1). Total reads aligned to genes were used in all subsequent analyses. DEGs were identified using the CLC Genomics Workbench Differential Expression for RNA-Seq tool with a cutoff value of > 2-fold and FDR *p*-value < 0.05. DEGs are listed in Supporting Information in Tables S2-S6 and the dataset is available under GEO accession number GSE111738. GO enrichment analysis of DEGs was performed using the Arabidopsis best BLAST match and agriGO, an agriculturally focused web based GO analysis program^65^. The singular enrichment analysis tool was used to identify GO terms that were significantly enriched (FDR *p*-value < 0.05) using the TAIR10 genome as the background (Table S7). All heatmaps were generated in R using the heatmap.2 function in the gplots CRAN library (http://cran.r-project.org/web/packages/gplots/index.html). For GO enrichment heatmaps, the FDR *p*-value was used. For gene expression heatmaps, Log2 (FPKM) values exported from the CLC Genomics Workbench were used with z-scaling by row.

### qRT-PCR validation of selected transcripts

RNA for qRT-PCR validation was isolated from immunopurified ribosomes of promoter::HF-RPL18 plants as previously described for three additional biological replicates collected from a different set of trees at the 2-week post vernalization time point. For these studies, 50 ng of isolated RNA was pretreated with RQ1 DNase (Promega, Madison, WI, USA), followed by reverse transcription using SuperScript III First-Strand Synthesis System and random hexamer primers (Invitrogen by Life Technologies, Carlsbad, CA, USA). SYBR green real-time qRT-PCR was performed in an ABI Prism 7100 (Applied Biosystems, Foster City, CA, USA). The 18S rRNA was chosen as an internal control for normalization. Primer sequences used for the amplification of all selected genes are provided in Table S8.

## Electronic supplementary material

Supplemental Material 1

Dataset 1

## Data Availability

The dataset is available under GEO accession number GSE111738.
