# Supplementary material for: Identification of phloem-associated translatome alterations during leaf development in Prunus domestica L
Source: Hortic Res. 2019 Feb 1;6:16. doi: 10.1038/s41438-018-0092-4 (PMC6355854; doi:10.1038/s41438-018-0092-4)
Supplement: Supplementary file 1 — Supplemental Material 1 [file 41438_2018_92_MOESM1_ESM.docx]

# **Supplementary Information**

# Article title: Identification of phloem associated translatome alterations during leaf development in Prunus domestica L.

Authors: Tamara D. Collum, Elizabeth Lutton, C. Douglas Raines, Christopher Dardick and James N. Culver

The following supplementary information is available for this article:

Fig. S1 RPL18 protein alignment.

Fig. S2 Principal component analysis of the plum translatome dataset.

Fig. S3 Non-transgenic control principal component analysis

Fig. S4 HF-RPL18 expression.

Fig. S5 Phloem enriched GO biological processes

Fig. S6 Validation of RNAseq data.

**
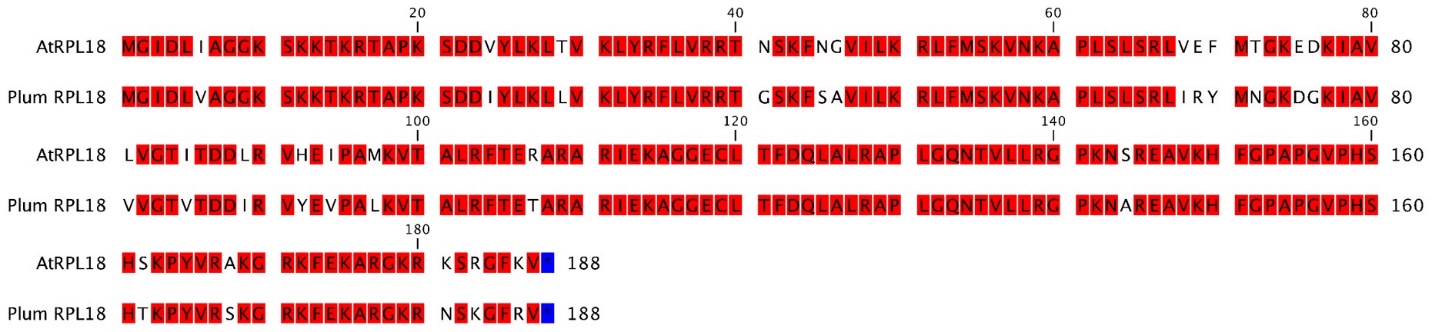
**

**Fig. S1** RPL18 protein alignment. *A. thaliana* RPL18 shares 87% amino acid identity and 95% similarity with plum RPL18. Conserved amino acid residues are highlighted in red.

**
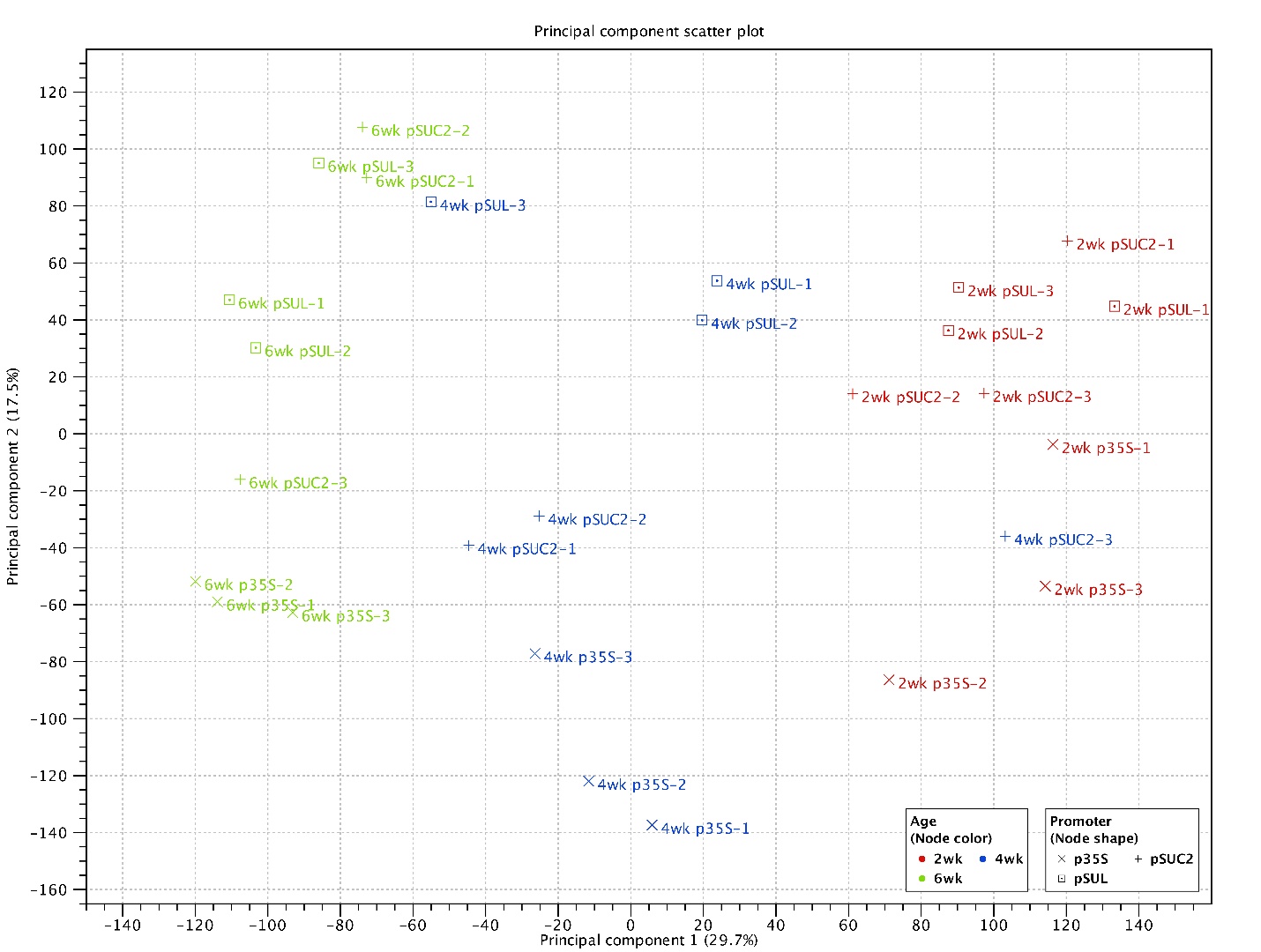
**

**Fig. S2** Principal component analysis of the plum translatome dataset. A plot of the RNA-seq data projected onto the first two principal components. Each spot represents a sample and color corresponds to leaf age (two weeks red, four weeks blue, six weeks green). Samples separated primarily on principal component 1 (29.7%) according to age and to a more limited extent on principal component 2 (17.5%) by construct.


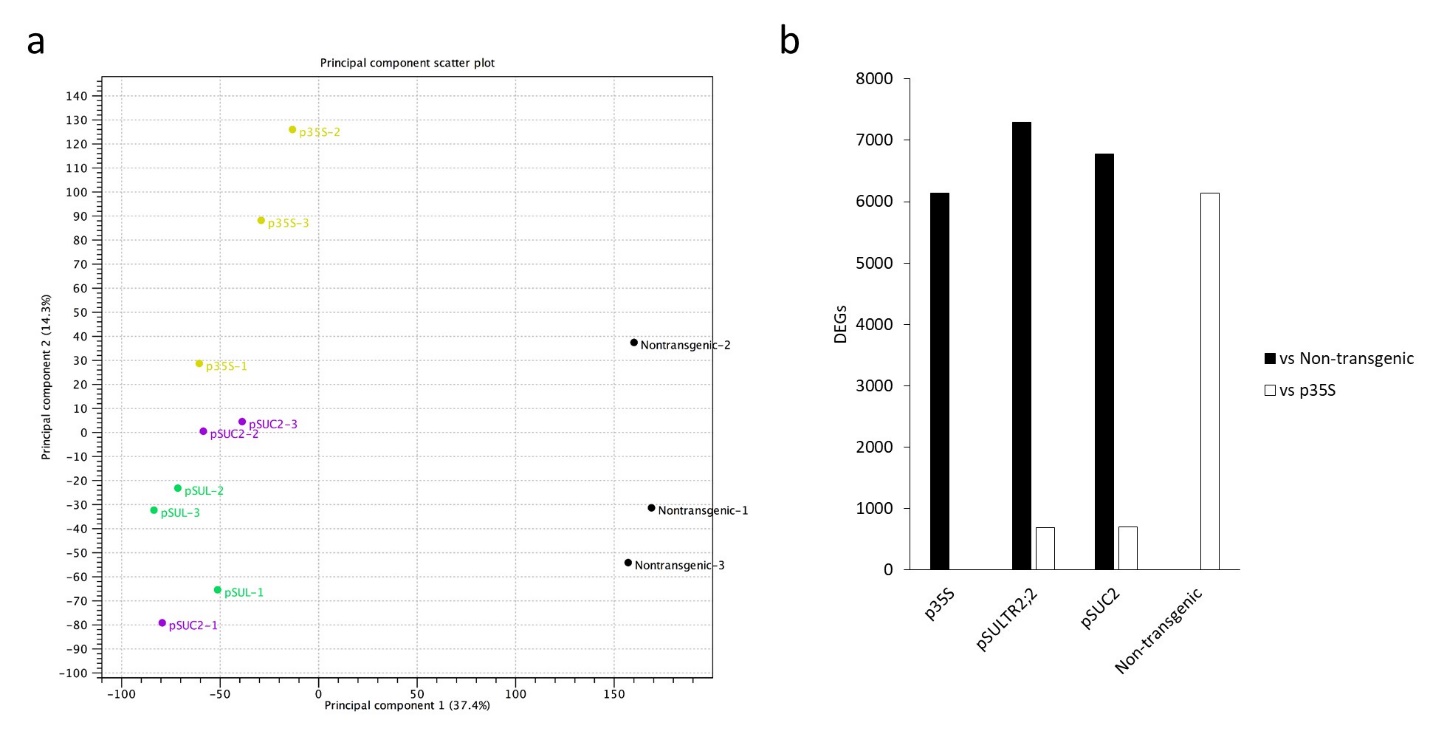


**Fig. S3** Non-transgenic control principal component analysis. (a) A plot of the RNA-seq data from the 2 week collection time point projected onto the first two principal components. Each spot represents a sample and color corresponds to promoter construct (p35S yellow, pSULTR2;2 green, pSUC2 purple, non-transgenic controls black). Non-transgenic samples separated primarily on principal component 1 (37.4%) while p35S samples separated to a more limited extent on principal component 2 (14.3%) from the two phloem promoter samples pSULTR2;2 and pSUC2. (b) Number of differentially expressed genes (DEGs) with a fold change >2 and FDR p-value < 0.05 during pairwise comparisons to non-transgenic controls (black bars) or p35S (white bars).

**
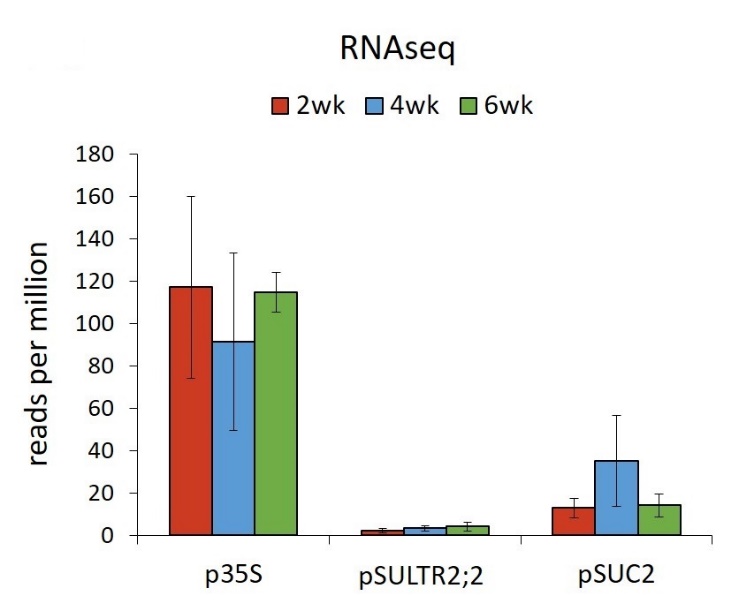
**

**Fig. S4** HF-RPL18 expression. Relative expression of HF-RPL18 transcripts measured by RNAseq. HF-RPL18 transcript levels were relatively consistent across biological replicates and time points. p35S samples had the greatest expression of HF-RPL18, followed by pSUC2 and then pSULTR2;2.

**
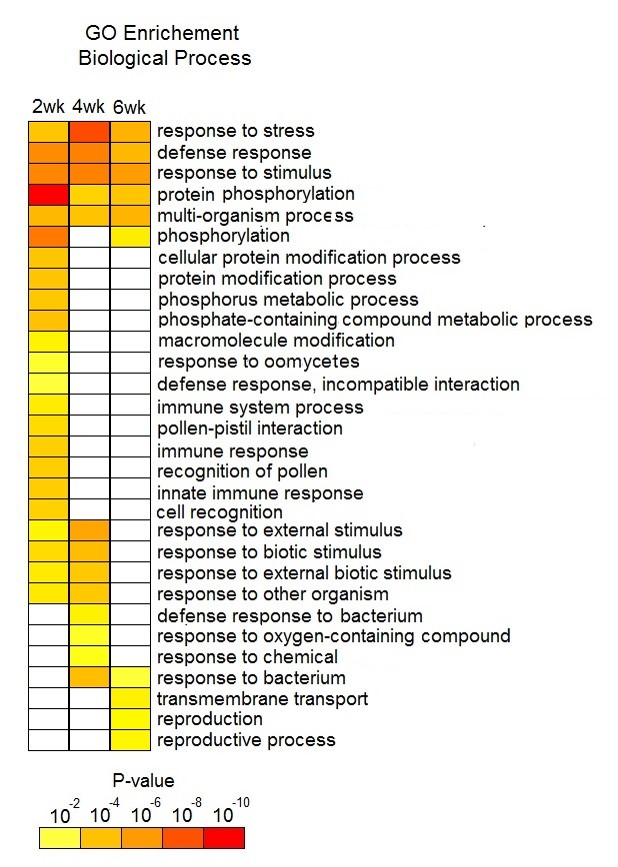
**

**Fig. S5** Phloem enriched GO biological processes. Heatmap showing significantly enriched (FDR p-value < 0.05) GO biological processes among shared genes enriched >2-fold in pSUC2 and pSULTR2;2 phloem translatomes compared to the p35S translatome at two, four, and six weeks post vernalization. Darker shading indicates higher statistical significance of GO term enrichment and white indicates no statistical significance.

**
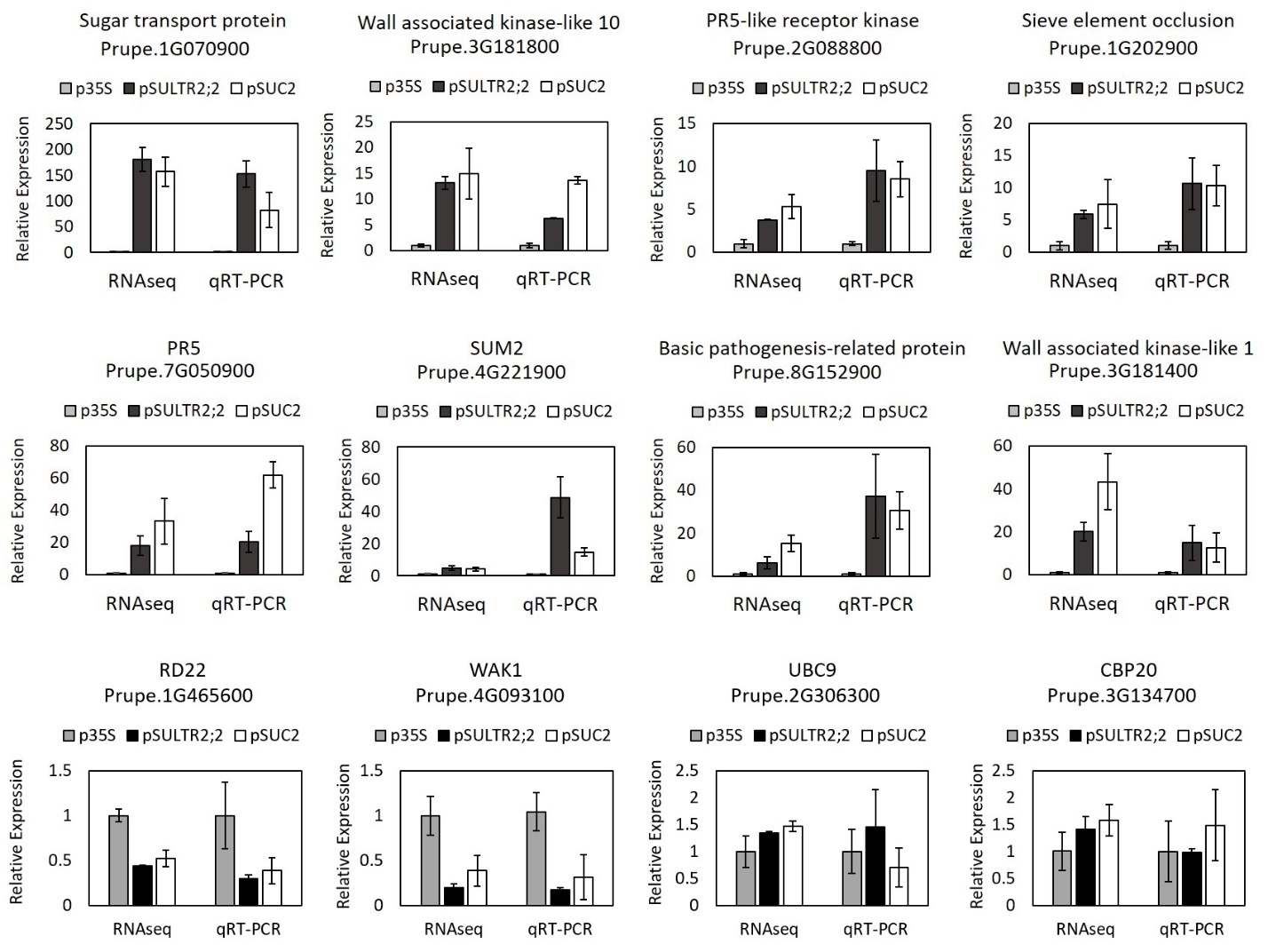
**

**Fig. S6** Validation of RNAseq data. Relative expression of selected transcripts at two weeks post vernalization. RNAseq data are mean and standard error of reads per kilobase per million mapped reads (RPKM) of three biological replicates. qRT–PCR data are mean and standard error of three additional translatome biological replicates collected from a different set of trees at the two-week post vernalization time point.
